# Emergency SARS-CoV-2 variants of concern: rapidly direct RT-qPCR detection without RNA extraction, clinical comparison, cost-effective, and high-throughput

**DOI:** 10.18632/aging.204095

**Published:** 2022-06-02

**Authors:** Bing-Heng Yang, Hsing-Yi Chung, Li-Ting Kao, Ming-Jr Jian, Chih-Kai Chang, Jung-Chung Lin, Kuo-Ming Yeh, Chien-Wen Chen, Ya-Sung Yang, Shan-Shan Hsieh, Sheng-Hui Tang, Cherng-Lih Perng, Feng-Yee Chang, Hung-Sheng Shang

**Affiliations:** 1 Division of Clinical Pathology, Department of Pathology, Tri-Service General Hospital, National Defense Medical Center, Taipei, Taiwan, ROC; 2 Department of Pharmacy Practice, Tri-Service General Hospital, National Defense Medical Center, Taipei, Taiwan, ROC; 3 Division of Infectious Diseases and Tropical Medicine, Department of Medicine, Tri-Service General Hospital, National Defense Medical Center, Taipei, Taiwan, ROC; 4 Division of Pulmonary and Critical Care Medicine, Department of Medicine, Tri-Service General Hospital, National Defense Medical Center, Taipei, Taiwan, ROC

**Keywords:** SARS-CoV-2 VOC, direct RT-qPCR, high-throughput, COVID-19, cost-effective diagnosis

## Abstract

Since the late 2020, the evolution of severe acute respiratory syndrome coronavirus 2 (SARS-CoV-2) variants of concern has been characterized by the emergence of spike protein mutations, and these variants have become dominant worldwide. The gold standard SARS-CoV-2 diagnosis protocol requires two complex processes, namely, RNA extraction and real-time reverse transcriptase polymerase chain reaction (RT-PCR). There is a need for a faster, simpler, and more cost-effective detection strategy that can be utilized worldwide, especially in developing countries. We propose the novel use of direct RT-qPCR, which does not require RNA extraction or a preheating step. For the detection, retrospectively, we used 770 clinical nasopharyngeal swabs, including positive and negative samples. The samples were subjected to RT-qPCR in the *N1* and *E* genes using two different thermocyclers. The limit of detection was 30 copies/reaction for *N1* and 60 copies/reaction for *E*. Analytical sensitivity was assessed for the developed direct RT-qPCR; the sensitivity was 95.69%, negative predictive value was 99.9%, accuracy of 99.35%, and area under the curve was 0.978. This novel direct RT-qPCR diagnosis method without RNA extraction is a reliable and high-throughput alternative method that can significantly save cost, labor, and time during the coronavirus disease 2019 pandemic.

## INTRODUCTION

Severe acute respiratory syndrome coronavirus-2 (SARS-CoV-2) has led to a global pandemic due to its rapid spread and the fatal progression of coronavirus disease (COVID-19) [1, 2]. Since December 2020, several biologically significant S protein mutations have been found to be associated with increased transmissibility, virulence, and diagnostic difficulties [3]. These variants are more transmissible and infectious than the original strains, leading to rapid spread and high mortality rates [4, 5].

The gold standard for SARS-CoV-2 screening relies on viral nucleic acid detection using real-time reverse transcription quantitative polymerase chain reaction (RT-qPCR) of the viral transport medium (VTM) collected from nasopharyngeal swabs of patients. The standard process includes RNA extraction and RT-qPCR [6–8]. This process is time and labor intensive, and expensive, creating a burden, especially in developing countries. Owing to the increased demand for screening SARS-CoV-2 variants of concern (SARS-CoV-2 VOCs), a new identification protocol that exhibits good performance and saves time and money is needed [9].

Direct RT-qPCR is a strategy to detect SARS-CoV-2 RNA directly from samples while bypassing RNA purification steps. This protocol could shorten the turnaround time of SARS-CoV-2 testing and reduce cost by eliminating the RNA extraction process [10]. A simplified protocol could offer a faster diagnosis of specimens to control the COVID-19 outbreak [11, 12]. In this study, we improved the protocol of RT-qPCR to ensure high sensitivity and specificity in the analysis of clinical specimens with high-throughput on LightCycler^®^ 96 (Roche Molecular Systems, Inc., USA) and LabTurbo™ AIO (LabTurbo Biotech Corporation, Inc., USA), which can perform 96 and 144 tests in one run, respectively [13, 14].

There is a need for fast and simple detection strategies of SARS-CoV-2 that can be utilized worldwide, especially in developing countries [15, 16]. Here, we developed a first-line screening method for SARS-CoV-2 that does not require RNA extraction and can be applied to different variants. We provide an alternative workflow for the molecular detection of SARS-CoV-2 VOCs; it is time-saving and cost-efficient, presents high-throughput, and is easy to operate.

## RESULTS

### SARS-CoV-2 specimen detection using conventional RT-qPCR and lab-developed direct RT-qPCR with commercial reagent

A total of 116 SARS-CoV-2 positive and 654 negative specimens were used to validate the performance of the lab-developed direct RT-qPCR. All specimens were confirmed using the conventional RT-qPCR method (with the RNA extraction protocol) as the control protocol, with the Ct values of positive SARS-CoV-2 specimens ranging from 11 to 34. The extracted RNA was analyzed using TIB VirSNiP Variant Kits to classify the variants. In 116 retrospectively positive specimens, 110 belonged to B.1.1.7, 3 to B.1.315, and 3 to B.1.617. The conventional PCR and lab-developed direct RT-qPCR of all samples that were classified as VOCs were performed (Supplementary Table 1).

### Comparative performance of lab-developed direct RT-qPCR on two real-time PCR machines

The positive samples were treated using the lab-developed direct RT-qPCR protocol on two real-time PCR machines (the LC96 and AIO). The Ct value of *N1* and *E* of each specimen was obtained using the lab-developed direct RT-qPCR and the conventional RT-qPCR (with extracted RNA protocol) as the reference method.

**Figure 1:**
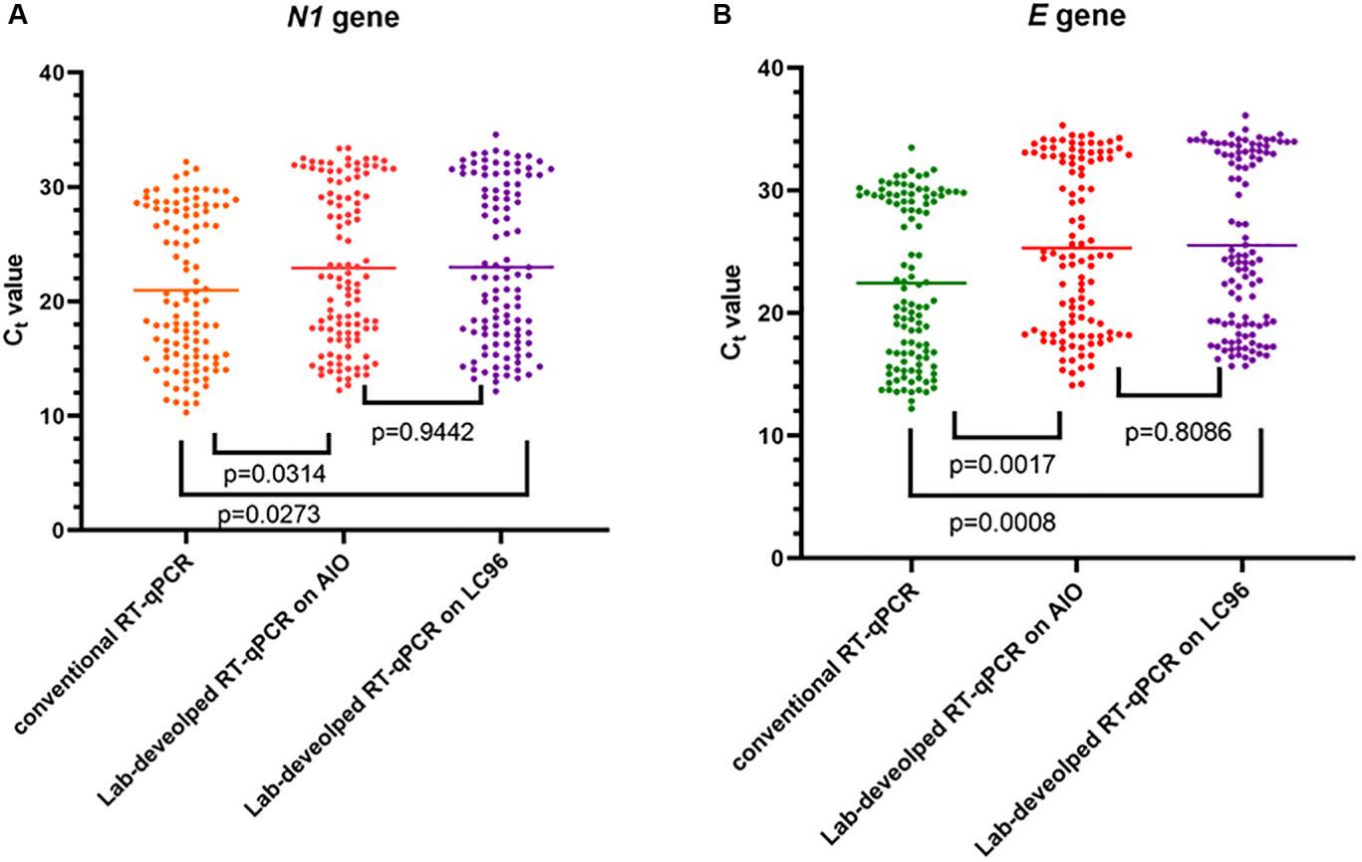
**Detection of SARS-CoV-2 using two methods on two different machines.** Data are depicted as scattered dot plots with stated mean values. Each dot represents one C_t_ value corresponding with one specimen and *p*-values were found using paired *t*-test. (**A**) Shows SARS-CoV-2 *N1* gene results. (**B**) Shows SARS-CoV-2 *E* gene results.

The distribution of the Ct values is shown in Figure 1. For the *N1* gene, the lab-developed direct protocol performed on both LC96 and LabTurbo AIO showed a higher Ct value than the conventional RT-qPCR with an average increase of 1.80 (*p* = 0.0273) and 1.65 (*p* = 0.0314), respectively. In addition, the Ct value examined using the lab-developed direct protocol showed similar patterns to conventional protocol in the *E* gene. For the *E* gene, the average Ct value increased by 2.53 (*p* = 0.0008) on LC96 and 2.91 (*p* = 0.0017) on LabTurbo AIO, respectively. Hence, the lab-developed direct RT-qPCR showed an increased Ct level for the *N1* and *E* genes compared with conventional RT-qPCR. For the *t*-test, *p* = 0.9442 for *N1* and *p* = 0.8086 for *E*.

**Figure 2:**
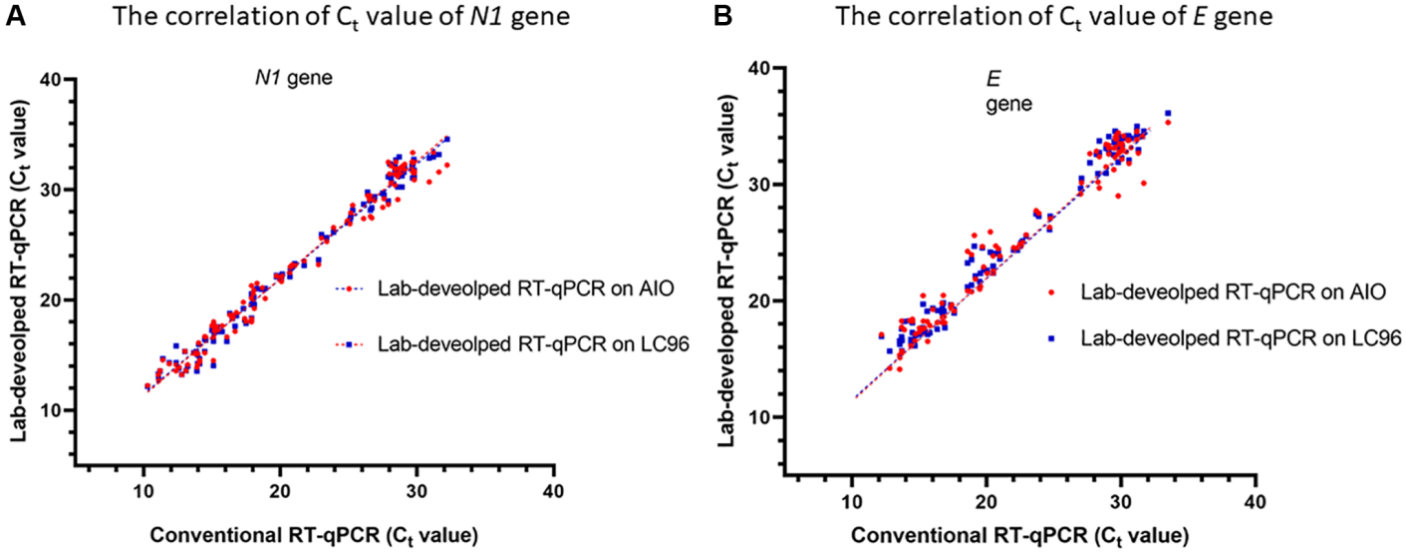
Correlation between cycle threshold values of *N1* (**A**) and *E* (**B**) genes using conventional RT- qPCR and lab-developed RT-qPCR on LC96 or AIO. The correlation coefficient of the analysis on LC96 is R^2^ = 0.9787 for *N1* gene and 0.9524 for *E* gene. The correlation coefficient of the analysis on AIO is R^2^ = 0.9850 of *N1* and 0.9798 of *E* gene.

The correlation between the Ct values of the samples analyzed using the two thermocyclers is shown in Figure 2. The Ct values of *N1* showed a high correlation on LabTurbo AIO (R^2^ = 0.9787) and LC96 (R^2^ = 0.9850). The *E* gene showed a similar performance to that of *N1*. Conversely, the Ct values of *E* showed a high correlation in both methods, with R^2^ = 0.9798 in LabTurbo AIO and R^2^ = 0.9524 in LC96. This correlation was also shown to be high for the positive samples. We also executed the conventional RT-qPCR and lab-developed direct RT-qPCR with two kinds of reagent on two different real-time PCR machines (LC96 and AIO). One reagent contained the primer and probe following the guidelines of US CDC and WHO (RT-PCR master mix with *N1* and *E*). For the other reagent, we followed the guidelines of WHO (RT-PCR master mix with *Rdrp* and *E*). The sequences are shown in Supplementary Table 2. We found that the different RT-PCR master mixes have concordance performance (Supplementary Table 3).

### Characteristics of the lab-developed direct RT-qPCR compared with those of conventional RT-qPCR with a commercial reagent to detect SARS-CoV-2

We tested all 770 samples using lab-developed direct and conventional RT-qPCR methods. The Ct values of *N1* and *E*, less than 35, were considered to be qualitative positive results. Table 1 summarizes the results of the lab-developed direct RT-qPCR compared with the conventional RT-qPCR method. The agreement between the lab-developed direct and conventional methods was 97.4%, which is considered high. The lab-developed direct RT-qPCR method presented a sensitivity of 95.69% and specificity of 100%. The negative likelihood ratio was 0.04. Analytical sensitivity was assessed for the lab-developed direct RT-qPCR method to obtain a sensitivity value of 95.69%, negative predictive value (NPV) of 99.9%, accuracy of 99.35%, and area under the receiver operating characteristics (ROC) curve (AUC) of 0.978. Our findings show that this novel direct RT-qPCR diagnosis method without RNA extraction is a reliable alternative method, which significantly saved cost, labor, and time, and presented high-throughput.

**Table 1:** Characteristics of lab-developed direct RT-qPCR compared to conventional RT-qPCR.

**(a) Agreement of the conventional RT-qPCR and lab-developed RT-qPCR**			
**Lab-developed RT-qPCR**	**Conventional RT-qPCR**		
	**Positive**	**Negative**	**Total**
Positive	111	0	111
Negative	5	654	659
Total	116	654	770
**(b) The qualitative results of conventional RT-qPCR and lab-developed RT-qPCR by agreement statistics**			
**Agreement Coefficient**	0.974 (CI = 0.952–0.994)		
**Accuracy**	99.35% (CI = 99.49–99.68%)		
**Sensitivity**	95.69% (CI = 90.23–98.59%)		
**Specificity**	100.00% (CI = 99.44–100.00%)		
**Positive predictive value**	100%		
**Negative predictive value**	99.24% (CI = 98.23–99.68%)		
**AUC ratio**	0.978 (CI = 0.957–1.000)		
**McNemar’s Test (*p*-value)**	0.65 (*p* < 0.001)		

### Analytical sensitivity of the lab-developed direct RT-qPCR assay

The limit of detection (LoD) of the lab-developed direct RT-qPCR was determined by testing 20 replicates of the SARS-CoV-2 RNA controls that were two-fold serially diluted to approximately the expected LoD. Using LC96, the LoD obtained for the 20 replicate tests was 30 copies/reaction for *N1*. In the AIO open system, the LoD was the same (Table 2).

**Table 2:** Assessment of limit of detection for lab-developed direct RT-qPCR on the real-time PCR machine.

**Real-time PCR machine**	**Gene target/fluorescent dye**	**No. of replicates detected as positive / No. of replicates tested at indicated of RNA control copies per reaction (percentage)**					
		**240**	**120**	**60**	**30**	**15**	**7.5**
LightCycler^®^ 96	*N1*/FAM	10/10 (100)	20/20 (100)	20/20 (100)	19/20 (95)	17/20 (85)	10/20 (50)
	*E*/HEX	10/10 (100)	20/20 (100)	20/20 (100)	17/20 (85)	12/20 (60)	3/20 (15)
LabTurbo AIO	*N1*/FAM	10/10 (100)	20/20 (100)	20/20 (100)	19/20 (95)	16/20 (80)	11/20 (55)
	*E*/HEX	10/10 (100)	20/20 (100)	20/20 (100)	16/20 (80)	12/20 (60)	3/20 (15)

### Analytical specificity of the conventional RT-qPCR and lab-developed direct RT-qPCR

To assess the analytical specificity of RT-qPCR, clinical specimens, or cell supernatants of known respiratory viruses, including influenza A and influenza B viruses, rhinovirus/enterovirus, respiratory syncytial virus, parainfluenza virus, adenovirus, and coronavirus NL96, were used. Both conventional and lab-developed direct RT-qPCR did not show cross-reactivity with other respiratory pathogens (Supplementary Table 4).

## DISCUSSION

Although SARS-CoV-2 was first discovered in late 2019, there is still a need for a refined screening test. SARS-CoV-2 continually mutates at the spike protein [17, 18]. A reliable and simple assay for COVID-19 detection is needed to combat the spread of the disease. There is also a need for molecular testing. However, there is a shortage of PCR reagents, viral RNA extraction kits, and even instruments. The problem is evident in developing countries and remote areas where the supply of reagents is insufficient.

Our method only needs real-time PCR machines, and therefore, the need for an extraction machine can be eliminated in first-line screening. According to the manufacturers of real-time PCR machines, the capacity of each batch could be expanded beyond the maximum capacity of RNA extraction instruments. The direct RT-qPCR developed in this study could be easily adopted in more resource-limited settings, including most of the developing areas that, at present, completely lack access to RNA extraction. We aimed to test cost-effective and high-throughput alternatives for the molecular detection of SARS-CoV-2.

We obtained good and consistent results, with our agreement coefficient reaching 0.97. There were 116 positive SARS-CoV-2 specimens, with the Ct values between 11 and 34. In SARS-CoV-2-positive specimens with a low viral load (Ct value greater than 30), the sensitivity was 77% (17/22). Besides these positive specimens, positive detection was not possible for five out of nine specimens. They belonged to SARS-CoV-2 specimens with a relatively low viral load, with Ct values ranging between 33 and 34 (Supplementary Table 1). According to our criteria for interpreting SARS-CoV-2-positive specimens, both genes should be positive, and the Ct value of the genes should be less than 35. For a wider interpretation of the positive results based on our criteria, the specimens were considered positive when any one of the genes was positive, leading to high sensitivity of the lab-developed direct RT-qPCR of SARS-CoV-2 specimens with a very low viral load (Ct values ranged from 34 to 35) of 80%. If the specimens will present a signal for just one gene. We suggest re-examining low-positive specimens using conventional RT-qPCR for increasing the sensitivity of this lab-developed direct RT-qPCR. After obtaining positive results with the lab-developed direct RT-qPCR, the specimens were retested using a conventional method according to the WHO guideline for SARS-CoV-2. Furthermore, in the 111 positive samples, a moderate correlation was observed, which indicates that although the Ct values were significantly increased, the two assays were correlated in the *N1* and *E* genes (Table 1 and Figure 2). The lab-developed direct protocol had a delayed Ct value similar to that of the conventional one. In the conventional RT-qPCR, an initial 500 μL of sample is eluted in 60 μL of elution buffer for the RNA extraction, resulting in approximately eight-fold increase in the concentration of the sample RNA. The lab-developed direct protocol showed good concordance with the conventional protocol, with an average delay in the Ct values of 1.80 for *N1* and 2.91 for *E* for the positive samples. The performance of the direct RT-qPCR developed in this study also proved to be effective in detecting SARS-CoV-2 in clinical samples with varying viral loads.

The main advantages of this protocol include the following: reducing the diagnostic test time from several hours to 1.5 h, increasing cost-effectiveness, easing the operation process, decreasing the probability of contamination, and easing the burden of extraction reagent supply. Our protocol increases the testing capacity during the period limited supplies. In another recent study to assess lab-developed direct RT-qPCR, only specific types of virus containers or other pretreatments were used [19, 20].

After a literature review, we chose nasopharyngeal swabs in the viral transport medium (VTM) as a template for lab-developed direct RT-qPCR, which showed better performance. Moreover, our data showed good consistency among SARS-CoV-2 VOCs. In 116 retrospective positive samples, 110 specimens belonged to B.1.1.7, 3 to B.1.315, and 3 to B.1.617. All five false-negative specimens belonged to B.1.1.7. We used a whole new gradient setting of the real-time PCR machines to achieve an increase in assay sensitivity. This lab-developed direct RT-qPCR assay could skip the RNA extraction step and has little effect on the sensitivity (Table 1). As mentioned earlier, only five specimens could not be detected using the new method, and the Ct values ranged from 34 to 35 (Supplementary Table 1). This finding may be caused by the inability to increase the specimen volume; therefore, this method is still applicable in the clinical setting. Our study focused on the lab-developed direct RT-qPCR applied to SARS-CoV-2 variants responsible for the current global pandemic.

## METHODS

### Study design and sample collection

Clinical nasopharyngeal swabs were collected in 2.5 mL of VTM. Retrospectively, upper respiratory tract specimens were collected from 116 SARS-CoV-2-positive and 654 SARS-CoV-2-negative individuals. All positive samples were confirmed by the Taiwan Centers for Disease Control and Prevention Central Laboratory [7, 14]. A total of 770 retrospective samples were assessed using two methods, conventional RT-qPCR with RNA extraction and our lab-developed direct RT-qPCR without RNA extraction at the same time. This retrospective study was registered on February 8, 2021 and approved by the Institutional Review Board of the Clinical and Genomic Research Committee at the Tri-Service General Hospital (approval no.: C2020205041). All methods were performed in accordance with the 1964 Declaration of Helsinki and other relevant guidelines and regulations.

### RNA extraction

For conventional RT-qPCR, the RNA from the upper respiratory tract specimens was extracted using the LabTurbo AIO platform. The clinical nasopharyngeal swabs were collected in 2.5 mL of VTM (LIBO Specimen Collection and Transport Swab Kits with Universal Transport Medium (New Taipei City, Taiwan)). The total viral nucleic acids were automatically extracted from 500 μL of samples to a final eluate volume of 60 μL using the LabTurbo Viral DNA/RNA Mini Kit (Cat. No. LVX480-500).

### RT-qPCR detection

For the two methods, we used two kinds of templates for RT-qPCR. The template volume used for the conventional RT-qPCR was 6 μL of RNA extracted from LabTurbo AIO. For the lab-developed direct RT-qPCR, we used 8 μL of VTM collected from nasopharyngeal swabs of the patients. A 25-μL RT-qPCR mixture contained the template as input material for the LabTurbo™ AIO COVID-19 RNA Testing Kit (Acov11240, Taipei, Taiwan) following the protocol of the commercial kit, which contains the primer for *N1*, *E*, and *RP*s (Supplementary Table 2). For *E*, we followed the WHO guidelines; whereas, for *N1* and *RP*, we followed the US CDC guidelines [7, 12]. The conventional RT-qPCR assays were performed under the following conditions: reverse transcription at 50°C for 10 min and initial denaturation at 95°C for 2 min, 40 cycles at 95°C for 5 s, and 58°C for 30 s. The lab-developed direct RT-qPCR assays were performed under the following conditions: reverse transcription at 46°C for 20 min and initial denaturation at 95°C for 2 min, 40 cycles at 95°C for 3 s, and 62°C for 15 s. For the lab-developed direct RT-qPCR, we used a PCR master mixture of a commercial kit, LabTurbo™ AIO COVID-19 RNA Testing Kit. We performed the lab-developed direct RT-qPCR assay on two different PCR thermocyclers—Roche LightCycler^®^ 96 System and LabTurbo AIO open system. A specimen was considered positive if the amplification curve crossed the threshold line within 35 cycles (Ct < 35).

### Analytical validation using RNA control

We used purified AMPLIRUN^®^ RNA controls (Vircell; Granada, Spain) of SARS-CoV-2 viral genes for absolute quantification. These controls were used to prepare a serial dilution panel with approximately 20 replicates, and the detailed protocol has been described previously [4]. We prepared dilutions of the RNA controls (240, 120, 60, 30, 15, and 7.5 copies/reaction) using nuclease-free water to assess the limit of detection (LoD). Using 8 μL of samples, we detected the performance of the lab-developed direct RT-qPCR on two different thermocyclers (LightCycler^®^ 96 and LabTurbo™ AIO). LoD was defined as a 95% probability of positive replicates.

### Evaluation of specificity

The specificity of the conventional RT-qPCR was evaluated using common upper respiratory tract viruses, as described in our earlier report [9]. For the lab-developed direct RT-qPCR, clinical samples or cell supernatants positive for influenza A and influenza B viruses, rhinovirus/Enterovirus, respiratory syncytial virus, parainfluenza virus, adenovirus, and coronavirus NL96 were obtained from the Taiwan CDC Viral Infection Contract Laboratory.

### Performance of the conventional RT-qPCR and lab-developed direct RT-qPCR using clinical samples

To evaluate the clinical performance of the lab-developed direct SARS-CoV-2 assay at varying viral concentrations, using the LC96 System and AIO open system, 116 positive specimens were selected to represent the full range of observed Ct values (11–34 cycles). Positive and negative agreements were calculated using two RT-qPCR methods, with the conventional RT-qPCR (with the extracted RNA protocol) as the reference method.

### Statistical analysis

For statistical analyses, data were collected and analyzed using SPSS version 19.0 (SPSS Inc.; Chicago, IL, USA) for Windows. Sensitivity, specificity, and positive and negative predictive value statistical measures were used to compare the conventional RT-qPCR results with the lab-developed direct RT-qPCR assay results. Boxplots and correlation graphs were used to show the distribution of Ct values between the methods.

### Detection of SARS-CoV-2 VOC

To screen for SARS-CoV-2 VOCs, we used VirSNiP SARS-CoV-2 spike N501Y, spike del H69/V70, spike K417N, and spike P681R (TIB Molbiol; Berlin, Germany); real-time RT-PCR post-melting curve analysis was used to detect the mutations in SARS-CoV-2-positive specimens. Here, we detected spike gene mutations on LightCycler 480 (Roche Molecular Systems, Inc., USA) according to the manufacturer’s instructions to confirm that the results were consistent with those of the rapid detection of SARS-CoV-2 VOCs.

## Supplementary Materials

Supplementary Table 1

Supplementary Tables 2-4
