# Supplementary material for: Emergency SARS-CoV-2 variants of concern: rapidly direct RT-qPCR detection without RNA extraction, clinical comparison, cost-effective, and high-throughput
Source: Aging (Albany NY). 2022 Jun 2;14(11):4624–33. doi: 10.18632/aging.204095 (PMC9217698; doi:10.18632/aging.204095)
Supplement: Supplementary Table 1 [file aging-14-204095-s001.docx]

**Supplementary Table 1. The C_t_ value of the 116 positive SARS-CoV-2 variant of concern in conventional RT-qPCR and lab-developed direct RT-qPCR on LightCycler® 96 and LabTurbo AIO.**

| Sample  serial No. | Conventional RT-qPCR | | | Lab-developed direct RT-qPCR | | | | | | | SARS-CoV-2 variant classification |
| --- | --- | --- | --- | --- | --- | --- | --- | --- | --- | --- | --- |
|  |  |  |  | | Roche LightCycler® 96 | | | LabTurbo^TM^ AIO open system | | |  |
|  | *N1* gene | *E* gene | interpretation | | *N1* gene | *E* gene | interpretation | *N1* gene | *E* gene | interpretation |  |
| W2021-01 | 18.00 | 19.55 | Positive | | 20.01 | 22.36 | Positive | 20.55 | 21.22 | Positive | B.1.315 |
| W2021-02 | 25.30 | 29.10 | Positive | | 28.12 | 34.11 | Positive | 28.59 | 33.37 | Positive | B.1.315 |
| W2021-03 | 26.40 | 28.90 | Positive | | 29.77 | 33.11 | Positive | 29.47 | 33.44 | Positive | B.1.315 |
| W2021-04 | 32.20 | 34.50 | Positive | | 34.58 | 34.88 | Positive | 32.22 | 34.32 | Positive | B.1.617 |
| W2021-05 | 20.15 | 22.31 | Positive | | 22.35 | 24.36 | Positive | 22.18 | 24.55 | Positive | B.1.617 |
| W2021-06 | 25.16 | 27.08 | Positive | | 27.52 | 30.51 | Positive | 27.89 | 30.15 | Positive | B.1.617 |
| W2021-07 | 33.80 | 34.52 | Positive | | 34.58 | n.d. | Negative | 33.89 | 36.11 | Negative | B.1.1.7 |
| W2021-08 | 20.02 | 21.00 | Positive | | 22.10 | 23.58 | Positive | 22.01 | 23.85 | Positive | B.1.1.7 |
| W2021-09 | 15.76 | 16.74 | Positive | | 17.15 | 18.95 | Positive | 17.65 | 18.55 | Positive | B.1.1.7 |
| W2021-10 | 28.46 | 29.47 | Positive | | 32.68 | 32.55 | Positive | 32.51 | 31.25 | Positive | B.1.1.7 |
| W2021-11 | 12.38 | 13.55 | Positive | | 15.85 | 16.45 | Positive | 13.58 | 14.11 | Positive | B.1.1.7 |
| W2021-12 | 12.38 | 13.55 | Positive | | 14.32 | 16.55 | Positive | 14.11 | 15.35 | Positive | B.1.1.7 |
| W2021-13 | 15.12 | 16.03 | Positive | | 17.69 | 19.36 | Positive | 18.01 | 19.25 | Positive | B.1.1.7 |
| W2021-14 | 29.04 | 30.08 | Positive | | 31.58 | 32.22 | Positive | 31.85 | 32.18 | Positive | B.1.1.7 |
| W2021-15 | 29.64 | 31.19 | Positive | | 32.00 | 34.61 | Positive | 31.82 | 34.51 | Positive | B.1.1.7 |
| W2021-16 | 29.64 | 31.19 | Positive | | 32.15 | 34.19 | Positive | 32.51 | 33.85 | Positive | B.1.1.7 |
| W2021-17 | 14.47 | 14.39 | Positive | | 15.33 | 16.66 | Positive | 15.21 | 16.11 | Positive | B.1.1.7 |
| W2021-18 | 14.47 | 14.39 | Positive | | 16.45 | 16.36 | Positive | 16.69 | 16.14 | Positive | B.1.1.7 |
| W2021-19 | 13.22 | 13.72 | Positive | | 13.99 | 15.69 | Positive | 14.12 | 15.51 | Positive | B.1.1.7 |
| W2021-20 | 15.18 | 16.91 | Positive | | 17.31 | 17.69 | Positive | 17.66 | 18.11 | Positive | B.1.1.7 |
| W2021-21 | 16.31 | 16.84 | Positive | | 18.79 | 19.11 | Positive | 18.65 | 18.61 | Positive | B.1.1.7 |
| W2021-22 | 18.69 | 20.06 | Positive | | 20.96 | 22.63 | Positive | 21.11 | 22.36 | Positive | B.1.1.7 |
| W2021-23 | 15.39 | 16.39 | Positive | | 17.56 | 19.15 | Positive | 17.25 | 18.15 | Positive | B.1.1.7 |
| W2021-24 | 13.86 | 15.23 | Positive | | 15.32 | 17.32 | Positive | 15.11 | 17.55 | Positive | B.1.1.7 |
| W2021-25 | 29.77 | 30.75 | Positive | | 32.74 | 33.95 | Positive | 32.47 | 33.15 | Positive | B.1.1.7 |
| W2021-26 | 26.53 | 28.18 | Positive | | 29.01 | 32.58 | Positive | 29.12 | 32.85 | Positive | B.1.1.7 |
| W2021-27 | 15.75 | 16.73 | Positive | | 17.13 | 19.32 | Positive | 17.66 | 18.12 | Positive | B.1.1.7 |
| W2021-28 | 14.54 | 14.74 | Positive | | 16.36 | 17.02 | Positive | 16.65 | 17.15 | Positive | B.1.1.7 |
| W2021-29 | 16.79 | 17.37 | Positive | | 18.58 | 19.65 | Positive | 18.85 | 19.39 | Positive | B.1.1.7 |
| W2021-30 | 24.92 | 27.01 | Positive | | 27.01 | 29.65 | Positive | 27.18 | 29.18 | Positive | B.1.1.7 |
| W2021-31 | 20.87 | 22.70 | Positive | | 22.98 | 25.13 | Positive | 23.18 | 25.01 | Positive | B.1.1.7 |
| W2021-32 | 16.55 | 17.42 | Positive | | 18.29 | 19.36 | Positive | 18.69 | 19.65 | Positive | B.1.1.7 |
| W2021-33 | 20.09 | 22.02 | Positive | | 22.05 | 24.35 | Positive | 21.68 | 24.55 | Positive | B.1.1.7 |
| W2021-34 | 11.09 | 12.81 | Positive | | 13.28 | 15.68 | Positive | 13.22 | 14.21 | Positive | B.1.1.7 |
| W2021-35 | 15.17 | 15.64 | Positive | | 17.02 | 17.11 | Positive | 17.20 | 16.51 | Positive | B.1.1.7 |
| W2021-36 | 15.35 | 16.34 | Positive | | 17.12 | 17.55 | Positive | 17.56 | 18.11 | Positive | B.1.1.7 |
| W2021-37 | 21.75 | 22.58 | Positive | | 23.15 | 24.96 | Positive | 23.55 | 24.69 | Positive | B.1.1.7 |
| W2021-38 | 13.96 | 15.03 | Positive | | 14.23 | 17.11 | Positive | 14.15 | 17.31 | Positive | B.1.1.7 |
| W2021-39 | 13.96 | 15.03 | Positive | | 15.03 | 17.21 | Positive | 15.12 | 17.65 | Positive | B.1.1.7 |
| W2021-40 | 19.73 | 20.50 | Positive | | 22.09 | 24.02 | Positive | 22.18 | 23.85 | Positive | B.1.1.7 |
| W2021-41 | 19.73 | 20.50 | Positive | | 22.23 | 22.98 | Positive | 22.18 | 22.55 | Positive | B.1.1.7 |
| W2021-42 | 16.50 | 17.60 | Positive | | 18.35 | 18.96 | Positive | 18.61 | 19.12 | Positive | B.1.1.7 |
| W2021-43 | 16.50 | 17.60 | Positive | | 18.31 | 19.21 | Positive | 18.33 | 19.14 | Positive | B.1.1.7 |
| W2021-44 | 20.73 | 22.51 | Positive | | 22.85 | 24.69 | Positive | 23.02 | 24.88 | Positive | B.1.1.7 |
| W2021-45 | 21.08 | 22.96 | Positive | | 23.31 | 25.56 | Positive | 23.13 | 25.65 | Positive | B.1.1.7 |
| W2021-46 | 18.07 | 19.18 | Positive | | 20.25 | 22.14 | Positive | 20.88 | 21.85 | Positive | B.1.1.7 |
| W2021-47 | 27.59 | 29.58 | Positive | | 29.52 | 34.58 | Positive | 29.18 | 32.32 | Positive | B.1.1.7 |
| W2021-48 | 15.00 | 15.31 | Positive | | 17.26 | 17.69 | Positive | 17.65 | 18.25 | Positive | B.1.1.7 |
| W2021-49 | 13.02 | 13.72 | Positive | | 15.33 | 16.58 | Positive | 15.35 | 15.65 | Positive | B.1.1.7 |
| W2021-50 | 23.01 | 24.75 | Positive | | 25.93 | 27.25 | Positive | 25.61 | 27.05 | Positive | B.1.1.7 |
| W2021-51 | 13.67 | 14.69 | Positive | | 15.22 | 16.15 | Positive | 15.18 | 16.55 | Positive | B.1.1.7 |
| W2021-52 | 14.02 | 15.40 | Positive | | 15.98 | 17.35 | Positive | 16.11 | 17.65 | Positive | B.1.1.7 |
| W2021-53 | 11.89 | 13.57 | Positive | | 14.32 | 16.25 | Positive | 14.23 | 15.11 | Positive | B.1.1.7 |
| W2021-54 | 15.10 | 16.80 | Positive | | 16.26 | 19.81 | Positive | 16.62 | 20.46 | Positive | B.1.1.7 |
| W2021-55 | 17.90 | 20.30 | Positive | | 19.58 | 24.16 | Positive | 18.28 | 25.91 | Positive | B.1.1.7 |
| W2021-56 | 26.60 | 29.70 | Positive | | 29.18 | 33.85 | Positive | 29.44 | 34.01 | Positive | B.1.1.7 |
| W2021-57 | 11.40 | 13.70 | Positive | | 14.68 | 16.88 | Positive | 14.56 | 17.73 | Positive | B.1.1.7 |
| W2021-58 | 28.40 | 29.10 | Positive | | 31.68 | 33.18 | Positive | 31.40 | 33.10 | Positive | B.1.1.7 |
| W2021-59 | 13.10 | 14.50 | Positive | | 13.60 | 17.25 | Positive | 13.59 | 18.42 | Positive | B.1.1.7 |
| W2021-60 | 10.30 | 13.70 | Positive | | 12.16 | 17.58 | Positive | 12.23 | 18.26 | Positive | B.1.1.7 |
| W2021-61 | 18.10 | 20.50 | Positive | | 19.58 | 22.39 | Positive | 19.23 | 22.38 | Positive | B.1.1.7 |
| W2021-62 | 18.30 | 19.70 | Positive | | 21.01 | 24.56 | Positive | 21.50 | 24.65 | Positive | B.1.1.7 |
| W2021-63 | 18.90 | 20.70 | Positive | | 21.05 | 24.07 | Positive | 20.13 | 24.70 | Positive | B.1.1.7 |
| W2021-64 | 23.90 | 27.70 | Positive | | 26.15 | 31.85 | Positive | 26.57 | 32.63 | Positive | B.1.1.7 |
| W2021-65 | 17.90 | 18.60 | Positive | | 20.32 | 23.25 | Positive | 20.15 | 24.24 | Positive | B.1.1.7 |
| W2021-66 | 16.10 | 18.90 | Positive | | 16.24 | 21.35 | Positive | 16.65 | 20.78 | Positive | B.1.1.7 |
| W2021-67 | 28.40 | 30.50 | Positive | | 31.66 | 34.18 | Positive | 31.90 | 33.80 | Positive | B.1.1.7 |
| W2021-68 | 29.80 | 29.10 | Positive | | 31.19 | 32.85 | Positive | 30.90 | 32.60 | Positive | B.1.1.7 |
| W2021-69 | 12.80 | 14.50 | Positive | | 13.25 | 18.11 | Positive | 13.23 | 18.22 | Positive | B.1.1.7 |
| W2021-70 | 25.10 | 28.90 | Positive | | 27.25 | 30.96 | Positive | 26.90 | 31.50 | Positive | B.1.1.7 |
| W2021-71 | 17.90 | 18.60 | Positive | | 18.37 | 21.18 | Positive | 18.02 | 20.85 | Positive | B.1.1.7 |
| W2021-72 | 26.70 | 29.60 | Positive | | 28.36 | 33.96 | Positive | 27.44 | 33.89 | Positive | B.1.1.7 |
| W2021-73 | 27.90 | 29.10 | Positive | | 28.96 | 32.92 | Positive | 28.66 | 33.20 | Positive | B.1.1.7 |
| W2021-74 | 27.90 | 29.60 | Positive | | 31.15 | 33.81 | Positive | 32.50 | 34.10 | Positive | B.1.1.7 |
| W2021-75 | 27.50 | 29.50 | Positive | | 29.69 | 33.65 | Positive | 28.40 | 33.10 | Positive | B.1.1.7 |
| W2021-76 | 28.00 | 29.70 | Positive | | 32.36 | 34.10 | Positive | 31.43 | 33.90 | Positive | B.1.1.7 |
| W2021-77 | 17.40 | 20.90 | Positive | | 18.19 | 24.11 | Positive | 18.31 | 24.46 | Positive | B.1.1.7 |
| W2021-78 | 29.70 | 31.60 | Positive | | 32.36 | 34.12 | Positive | 33.36 | 34.10 | Positive | B.1.1.7 |
| W2021-79 | 28.10 | 29.90 | Positive | | 31.05 | 33.08 | Positive | 30.60 | 32.80 | Positive | B.1.1.7 |
| W2021-80 | 29.80 | 30.10 | Positive | | 31.76 | 33.16 | Positive | 31.67 | 33.10 | Positive | B.1.1.7 |
| W2021-81 | 33.90 | 34.40 | Positive | | n.d. | 34.69 | Negative | n.d. | n.d. | Negative | B.1.1.7 |
| W2021-82 | 22.80 | 23.70 | Positive | | 23.65 | 27.47 | Positive | 23.20 | 27.74 | Positive | B.1.1.7 |
| W2021-83 | 31.20 | 30.60 | Positive | | 32.98 | 34.15 | Positive | 33.40 | 33.80 | Positive | B.1.1.7 |
| W2021-84 | 29.80 | 30.10 | Positive | | 31.05 | 33.19 | Positive | 31.50 | 32.90 | Positive | B.1.1.7 |
| W2021-85 | 26.10 | 28.40 | Positive | | 28.68 | 33.73 | Positive | 27.38 | 29.69 | Positive | B.1.1.7 |
| W2021-86 | 28.40 | 29.80 | Positive | | 32.08 | 33.61 | Positive | 31.90 | 32.60 | Positive | B.1.1.7 |
| W2021-87 | 28.90 | 30.10 | Positive | | 31.25 | 34.06 | Positive | 32.10 | 33.50 | Positive | B.1.1.7 |
| W2021-88 | 26.90 | 28.40 | Positive | | 29.36 | 32.73 | Positive | 29.03 | 32.37 | Positive | B.1.1.7 |
| W2021-89 | 29.70 | 31.30 | Positive | | 31.06 | 32.98 | Positive | 31.60 | 32.70 | Positive | B.1.1.7 |
| W2021-90 | 14.10 | 15.30 | Positive | | 14.66 | 19.69 | Positive | 14.55 | 20.44 | Positive | B.1.1.7 |
| W2021-91 | 12.60 | 14.30 | Positive | | 13.82 | 18.28 | Positive | 13.90 | 18.30 | Positive | B.1.1.7 |
| W2021-92 | 13.90 | 15.80 | Positive | | 13.55 | 17.26 | Positive | 13.89 | 17.88 | Positive | B.1.1.7 |
| W2021-93 | 17.50 | 18.90 | Positive | | 17.89 | 23.54 | Positive | 18.06 | 23.97 | Positive | B.1.1.7 |
| W2021-94 | 28.60 | 29.90 | Positive | | 31.28 | 33.74 | Positive | 31.20 | 33.47 | Positive | B.1.1.7 |
| W2021-95 | 28.40 | 29.70 | Positive | | 31.55 | 33.84 | Positive | 30.42 | 34.28 | Positive | B.1.1.7 |
| W2021-96 | 29.10 | 29.80 | Positive | | 32.13 | 34.34 | Positive | 32.30 | 34.43 | Positive | B.1.1.7 |
| W2021-97 | 28.70 | 30.20 | Positive | | 31.39 | 33.98 | Positive | 31.93 | 34.16 | Positive | B.1.1.7 |
| W2021-98 | 26.60 | 28.30 | Positive | | 28.15 | 30.93 | Positive | 27.57 | 30.20 | Positive | B.1.1.7 |
| W2021-99 | 11.10 | 13.90 | Positive | | 12.98 | 18.22 | Positive | 12.67 | 17.50 | Positive | B.1.1.7 |
| W2021-100 | 28.70 | 29.60 | Positive | | 32.98 | 33.28 | Positive | 31.50 | 33.20 | Positive | B.1.1.7 |
| W2021-101 | 16.70 | 19.10 | Positive | | 17.61 | 24.69 | Positive | 17.16 | 25.61 | Positive | B.1.1.7 |
| W2021-102 | 28.90 | 30.40 | Positive | | 30.25 | 34.00 | Positive | 32.10 | 32.80 | Positive | B.1.1.7 |
| W2021-103 | 20.70 | 23.90 | Positive | | 22.12 | 27.25 | Positive | 22.49 | 27.52 | Positive | B.1.1.7 |
| W2021-104 | 28.10 | 29.80 | Positive | | 32.25 | 31.90 | Positive | 32.17 | 29.00 | Positive | B.1.1.7 |
| W2021-105 | 15.10 | 16.70 | Positive | | 14.05 | 19.11 | Positive | 14.50 | 19.80 | Positive | B.1.1.7 |
| W2021-106 | 14.10 | 15.60 | Positive | | 14.22 | 19.11 | Positive | 14.40 | 18.20 | Positive | B.1.1.7 |
| W2021-107 | 17.90 | 19.50 | Positive | | 20.55 | 21.65 | Positive | 21.30 | 21.00 | Positive | B.1.1.7 |
| W2021-108 | 30.90 | 31.20 | Positive | | 32.88 | 34.98 | Positive | 30.70 | 34.58 | Positive | B.1.1.7 |
| W2021-109 | 33.50 | 34.80 | Positive | | 33.58 | n.d. | Negative | 34.98 | n.d. | Negative | B.1.1.7 |
| W2021-110 | 33.70 | 34.10 | Positive | | 35.65 | n.d. | Negative | 36.15 | n.d. | Negative | B.1.1.7 |
| W2021-111 | 17.30 | 19.80 | Positive | | 19.21 | 22.65 | Positive | 19.80 | 22.90 | Positive | B.1.1.7 |
| W2021-112 | 31.60 | 31.70 | Positive | | 33.18 | 34.58 | Positive | 31.60 | 30.10 | Positive | B.1.1.7 |
| W2021-113 | 11.20 | 12.20 | Positive | | 13.55 | 16.95 | Positive | 13.60 | 17.10 | Positive | B.1.1.7 |
| W2021-114 | 23.40 | 24.70 | Positive | | 25.65 | 26.13 | Positive | 25.30 | 26.30 | Positive | B.1.1.7 |
| W2021-115 | 28.60 | 30.60 | Positive | | 30.25 | 32.08 | Positive | 29.10 | 31.80 | Positive | B.1.1.7 |
| W2021-116 | 33.80 | 34.50 | Positive | | n.d. | 34.88 | Negative | 36.83 | 34.94 | Negative | B.1.1.7 |

n.d. indicated not detected.
