# Supplementary material for: Emergency SARS-CoV-2 variants of concern: rapidly direct RT-qPCR detection without RNA extraction, clinical comparison, cost-effective, and high-throughput
Source: Aging (Albany NY). 2022 Jun 2;14(11):4624–33. doi: 10.18632/aging.204095 (PMC9217698; doi:10.18632/aging.204095)
Supplement: Supplementary Tables 2-4 [file aging-14-204095-s002.pdf]

SUPPLEMENTARY TABLES

Supplementary Table 2. Primer and probe sequences used in the conventional RT-qPCR and lab-developed RT-qPCR.

**LabTurbo™ AIO COVID-19 RNA Testing Kit**			
**Primer name**	**Target gene**	**Sequence (5′→3′)**	**References**
E_Sarbeco_F		ACAGGTACGTTAATAGTTAATAGCGT	
E_Sarbeco_R	*E* gene	ATATTGCAGCAGTACGCACACA	[7]
E_Sarbeco_P		/5HEX/ACACTAGCC/ZEN/ATCCTTACTGCGCTTCG/3IABkFQ/	
2019-nCoV_N1-F		GACCCCAAAATCAGCGAAAT	
2019-nCoV_N1-R	*N1* gene	TCTGGTTACTGCCAGTTGAATCTG	[7]
2019-nCoV_N1-P		/56-FAM/ACCCCGCAT/ZEN/TACGTTTGGTGGACC/3IABkFQ/	
RP-F		AGATTTGGACCTGCGAGCG	
RP-R	*RNase P* gene	GAGCGGCTGTCTCCACAAGT	[7]
RP-P		/5Cy5/TTCTGACCT/TAO/GAAGGCTCTGCGCG/3IAbRQSp/	
**WHO**			
**Primer name**	**Target gene**	**Sequence (5′→3′)**	**References**
RdRP_SARSr-F2		GTGARATGGTCATGTGTGGCGG	
RdRP_SARSr-R1	*RdRP gene*	CARATGTTAAASACACTATTAGCATA	[14]
RdRP_SARSr-P2		FAM-CAGGTGGAACCTCATCAGGAGATGC-BBQ	
E_Sarbeco_F		ACAGGTACGTTAATAGTTAATAGCGT	
E_Sarbeco_R	*E* gene	ATATTGCAGCAGTACGCACACA	[14]
E_Sarbeco_P		/5HEX/ACACTAGCC/ZEN/ATCCTTACTGCGCTTCG/3IABkFQ/	
RP-F		AGATTTGGACCTGCGAGCG	
RP-R	*RNase P* gene	GAGCGGCTGTCTCCACAAGT	[7]
RP-P		/5Cy5/TTCTGACCT/TAO/GAAGGCTCTGCGCG/3IAbRQSp/	

Supplementary Table 3. Positive and negative agreement of conventional RT-qPCR versus lab-developed direct RT-qPCR used by COVID-19 RNA reagent and WHO.

		Conventional RT-qPCR				Lab-developed Direct RT-qPCR			
		COVID-19 RNA kit		WHO		COVID-19 RNA kit		WHO	
		N1	***E***	***Rdrp***	***E***	***N1***	***E***	***Rdrp***	***E***
Total positive (*n*)		116		116		111		111	
Ct value (*n*)	High (<20)	55	48	55	48	46	38	43	36
	Medium (20–29)	51	47	52	47	35	29	37	33
	Low (30–35)	9	22	8	22	33	45	32	42
Total negative (*n*)		654		654		659		659	

Supplementary Table 4. Cross-reaction of conventional RT-qPCR and lab-developed RT-qPCR with known respiratory viruses in clinical samples/cell supernatants.

**Viral pathogens**	**Convention RT-qPCR**	**Direct RT-qPCR**
Influenza A (H1)	Not detected	Not detected
Influenza A (H3)	Not detected	Not detected
Influenza A (H1N1)	Not detected	Not detected
Influenza B	Not detected	Not detected
Rhinovirus/ Enterovirus	Not detected	Not detected
Respiratory syncytial virus	Not detected	Not detected
Parainfluenza 1 virus	Not detected	Not detected
Parainfluenza 2 virus	Not detected	Not detected
Parainfluenza 3 virus	Not detected	Not detected
Adenovirus	Not detected	Not detected
Coronavirus NL96	Not detected	Not detected
